# Supplementary material for: Body image, obesity, and sexual coercion: Impacts on depression among students at a Nigerian university
Source: PLoS One. 2025 Jun 17;20(6):e0319308. doi: 10.1371/journal.pone.0319308 (PMC12173399; doi:10.1371/journal.pone.0319308)
Supplement: S1 Table — (DOCX) [file pone.0319308.s001.docx]

Supplementary Table 1.

Distribution of Study Participants by Academic Year

| Academic Year | Count | Percentage (%) |
| --- | --- | --- |
| Year 1 | 24 | 4.5 |
| Year 2 | 185 | 34.3 |
| Year 3 | 177 | 32.8 |
| Year 4 | 65 | 12.1 |
| Year 5 | 58 | 10.8 |
| Year 6 | 27 | 5 |
| Postgraduate | 3 | 0.6 |

*This table presents the distribution of the study population (N=501) across academic years, demonstrating the proportional representation achieved through stratified random sampling based on academic level. The stratification ensured inclusion of students from Year 1 to postgraduate levels in alignment with the study methodology.*
