# Supplementary material for: Body image, obesity, and sexual coercion: Impacts on depression among students at a Nigerian university
Source: PLoS One. 2025 Jun 17;20(6):e0319308. doi: 10.1371/journal.pone.0319308 (PMC12173399; doi:10.1371/journal.pone.0319308)
Supplement: S2 Table — (DOCX) [file pone.0319308.s002.docx]

Supplementary Table 2.

Distribution of Study Participants by Faculty

| Faculty | Count | Percentage |
| --- | --- | --- |
| Medicine & Health Sciences | 222 | 41.2 |
| Law | 106 | 19.7 |
| Other | 92 | 17.1 |
| Engineering | 76 | 14.1 |
| Computing & IT | 30 | 5.6 |
| Social & Management Sciences | 9 | 1.7 |
| Science | 4 | 0.7 |

*This table illustrates the distribution of participants across various faculties within the university. The stratification by faculty reflects the methodological approach to ensure diverse representation from different academic disciplines, supporting the generalizability of findings within the institutional context.*
